# The necessity for prompt identification of Sjögren syndrome in instances of unexplained heart failure: A case report

**DOI:** 10.1097/MD.0000000000042451

**Published:** 2025-05-16

**Authors:** Jiao Wu, Hongcai Zhang, Wen Xie, Mingjun Han, Qian Nie

**Affiliations:** a Eye School of Chengdu University of TCM, Chengdu University of Traditional Chinese Medicine, Chengdu, Sichuan Province, China; b Department of Cardiology, Hospital of Chengdu University of Traditional Chinese Medicine, Chengdu, Sichuan Province, China.

**Keywords:** antinuclear antibodies, heart failure, immunosuppressants, primary, Sjögren’s syndrome

## Abstract

**Rationale:**

Sjögren’s syndrome (SS) is a highly phenotypically diverse autoimmune disorder. It has been reported that 30% to 40% of patients with SS experience systemic complications, including peripheral neuropathies, interstitial lung disease, interstitial nephritis, and lymphoma. To date, SS presenting with heart failure (HF) as the primary manifestation remains exceedingly rare.

**Patient concerns:**

We report presents a 70-year-old female patient, devoid of any cardiovascular risk factors, who experienced recurrent episodes of HF.

**Diagnoses:**

During the course of treatment, SS was suspected based on her medical history. This suspicion was subsequently confirmed through elevated serum autoimmune antibody levels and a labial gland biopsy.

**Interventions:**

The implementation of standard HF management in conjunction with an immunosuppressive regimen.

**Outcomes:**

At the one-year follow-up, the patient had not been readmitted for HF.

**Lessons:**

This case underscores the critical importance of early identification of HF attributable to SS and highlights the efficacy of combining immunosuppressive therapy with conventional HF treatment as the optimal approach for managing such cases.

## 1. Introduction

Sjögren’s syndrome (SS) is a multifaceted, chronic autoimmune disorder affecting multiple systems, primarily characterized by lymphocytic infiltration of exocrine glands, notably the salivary and lacrimal glands, leading to symptoms such as xerostomia, xerophthalmia, fatigue, and arthralgia.^[1]^ SS is a phenotypically varied autoimmune disorder that can be divided to primary Sjögren’s syndrome (pSS) and secondary SS, which is associated with other connective tissue disease, such as rheumatoid arthritis, systemic lupus erythematosus and systemic sclerosis in some instances. A global epidemiological study has indicated that the prevalence of SS ranges from 0.01% to 0.05%, with a marked predominance in females aged 30 to 50 years, reflected in a male-to-female ratio of approximately 1:14.^[2]^ In the majority of instances, SS primarily affects the exocrine glands. Nevertheless, approximately 30% to 40% of patients may present with extraglandular manifestations, including those affecting the thyroid, lungs, gastrointestinal tract, hematological system, kidneys, skin, and both central and peripheral nervous systems.^[3]^ Notably, while cardiac involvement in SS can manifest as conduction block, autoimmune myocarditis, cardiomyopathy, and pericardial effusion.^[4]^ SS presenting with heart failure (HF) as the primary manifestation remains exceedingly rare, although a case of secondary SS complicated by HF was first documented in 2021.^[5]^ But up to date, there are extremely few studies described the eventual heart failure caused by the pSS in the literature.

In this report, we present a case involving a 70-year-old female patient who experienced recurrent episodes of unexplained HF as the primary clinical manifestation, despite having no prior history of cardiovascular disease. Ultimately, she was diagnosed with pSS-induced HF according to the American College of Rheumatology and the European Alliance of Associations for Rheumatology (ACR/EULAR)-approved diagnostic criteria of pSS.^[6]^ The implementation of standard HF management in conjunction with an immunosuppressive regimen resulted in a favorable clinical outcome for the patient. This case underscores the critical importance of early identification of HF attributable to pSS and highlights the efficacy of combining immunosuppressive therapy with conventional HF treatment as the optimal approach for managing such cases.

## 2. Case presentation

A 70-year-old female patient were admitted to hospital for a 6-year history of respiratory distress, bilateral lower-limb edema persisting for 12 months, and aggravation of chest tightness and dyspnea for 10 days. In 2018，she visited to a local hospital due to respiratory system symptom, especially cough, chest tightness, shortness of breath, and dyspnea. She had a history of hypothyroidism but no reported history of hypertension, diabetes, or coronary artery disease. A chest computed tomography scan revealed interstitial pneumonia, but she refused further thorough clinical examination and systematic treatment at the time. Subsequently, she has had recurrent respiratory system symptoms between 2018 and 2023. During the past 12 months，the patient condition aggravated further, and she presented with paroxysmal nocturnal dyspnea, bilateral lower limb depression and edema, which suggested HF symptoms.

**Figure 1.**
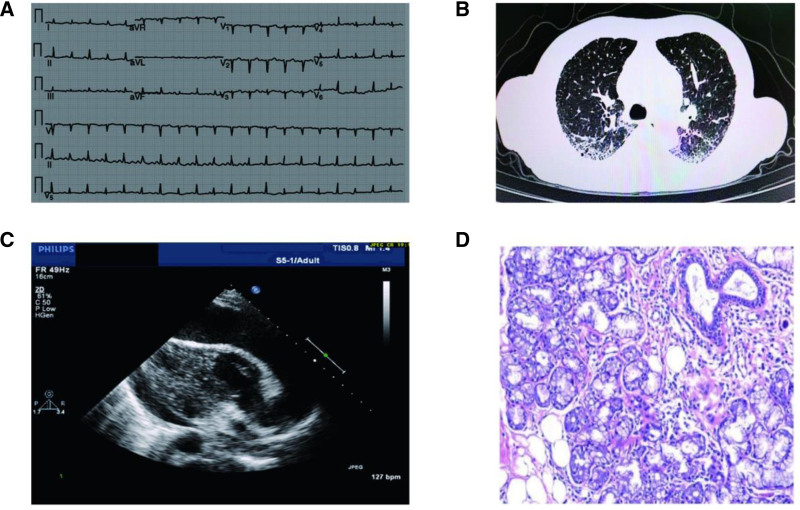
Examination results of the patient. (A) ECG shows sinus tachycardia with low voltage. (B) Chest CT indicates bilateral pulmonary interstitial fibrosis with superimposed infection. (C) Echocardiogram reveals a 45% left ventricular ejection fraction, moderate to large pericardial effusion, cardiac dilation, and pulmonary hypertension. (D) Labial gland biopsy shows small salivary gland tissues with significant lymphocyte and plasma cell infiltration. CT = computed tomography, ECG = electrocardiogram.

After admission, a physical examination of the patient were recorded as follows: a body temperature of 37.4°C, a heart rate of 112 beats/min, and a blood pressure of 80/47 mm Hg. A thorough physical examination revealed positive liver neck reflux, pronounced wet murmurs in both lungs, an enlarged cardiac silhouette, and distant heart sounds. Furthermore, significant edema was observed in both lower extremities. Then, we performed a urgent hematologic review for the patient, which included routine blood test, myocardial enzyme spectrum, C-reactive protein, procalcitonin, liver and kidney function tests, electrolyte assessment, and biochemistry tests (Table 1). Through laboratory analysis, it was determined that the patient exhibited a significant increase in white blood cell count (14.32 × 10^9^/L), alongside elevated cardiac biomarkers, including cardiac troponin I (0.7 ng/mL; reference value, <0.01 ng/mL), creatine kinase-isoenzyme (30 U/L), and N-terminal pro-brain natriuretic peptide (25,420 pg/mL). Additionally, the patient demonstrated signs of hepatic dysfunction, as evidenced by elevated serum levels of hyaluronic acid (234.20 ng/mL), laminin (53.11 ng/mL), type IV collagen (50.94 ng/mL), glycocholic acid hydrate (5.24 µg/mL), and the N-terminal peptide of type III procollagen (153.80 ng/mL). Furthermore, thyroid dysfunction was indicated by an increased thyroid stimulating hormone level (12.44 µIU/mL) and altered thyroid hormone concentration (64.88 nmol/L). Notably, the patient renal function remains within normal parameters. Electrocardiogram findings indicated sinus tachycardia with low voltage across all leads (Fig. 1A). The most recent chest computed tomography scan reveals evidence of chronic bronchitis, bilateral pulmonary interstitial fibrosis with superimposed infection, and mild to moderate pleural effusion (Fig. 1B). Furthermore, the cardiac echocardiogram results showed that the patient left ventricular ejection fraction was 45%, accompanied by moderate to large pericardial effusion, cardiac dilation, heart enlargement, and pulmonary hypertension (Fig. 1C). Thus, the patient was diagnosed with acute HF, hypothyroidism, and pulmonary infection.

**Table 1:** Laboratory tests of the patient.

Hematological tests	Results of the patient	Reference value
Routine blood test		
White blood cell count (×10^9^/L)	14.32	3.5–9.5
Neutrophil count (×10^9/^L)	9.14	1.8–6.3
Lymphocyte count (×10^9^/L)	3.81	1.1–3.2
Monocyte count (×10^9^/L)	1.31	0.1–0.9
Hemoglobin (g/L)	141	130–175
Thyroid function test		
Thyroid stimulating hormone (uTU/mL)	12.44	0.27–5.00
Thyroid hormones (nmol/L)	64.88	66–181
Triiodothyronine (nmol/L)	0.96	1.3–3.1
Free thyroxine (pmol/L)	14.71	12–22
Thyroglobulin (ng/mL)	35.86	3.5–77
Thyroglobulin antibodies	0.59	2.1–4.0 weakly positive; >4 positive
Antithyroid peroxidase antibody (IU/mL)	12.87	<34
Thyrotropin receptor antibody (IU/L)	1.11	<1.75
Indicators of hepaticfibrosis		
Hyaluronicacid (ng/mL)	234.20	0–100
Laminin (ng/mL)	53.11	0.51–50
Collagen type IV (ng/mL)	50.940	0.021–30
Cholyglycine (ug/mL)	5.24	0–2.7
Myocardial enzyme spectrum		
cTNI (ng/mL)	0.7	<0.01
CK-MB (U/L)	30	
NT-proBNP (pg/mL)	25,420	
Liver function tests		
Total protein (g/L)	68.9	65–85
Albumin (g/L)	30.7	40–55
Globulin (g/L)	38.2	20–40
Globulin ratio	0.8	1.2–2.4
Alanine aminotransferase (U/L)	16	7–40
Kidney function tests		
Urea (U/L)	6.21	3.1–8.8
Creatinine (μmol/L)	68.5	41–81
Uric acid (μmol/L)	425	142–339
Electrolyte		
Potassium (mmol/L)	3.64	3.5–5.3
Sodium (mmol/L)	135.9	137–147
Chlorine (mmol/L)	104.7	99–110
Calcium (mmol/L)	2.06	2.11–2.52
Magnesium (mmol/L)	0.81	0.75–1.02
Autoimmune antibody spectrum		
Antinuclear antibodies (ANA)	Positive	Negative (<100)
Anti-double-stranded DNA antibody	Negative	Negative (<10)
ANA (karyotype)		
Nuclear particle type	1:3200 (+++)	<1:00
Cytoplasmic granule type	>1000 (++)	<1:00
Anti-nRNP antibody	Negative	Negative
Anti-SM antibody	Negative	Negative
Anti-SSA antibody	Positive	Negative
Anti-SSB antibody	Weakly Positive	Negative
Anti-ScL-70	Negative	Negative
Anti-Jo-1	Negative	Negative

ANA = antinuclear antibodies, cTNI = cardiac troponin I, CK-MB = creatine kinase-isoenzyme, DNA = deoxyribonucleic acid, nRNP = nuclear ribonucleoprotein, NT-proBNP = N-terminal pro-brain natriuretic peptide, SM = smith, SSA = Sjögren's syndrome A, SSB = Sjögren's syndrome B.

Subsequently, we administered a comprehensive treatment regimen to the patient, which included cefoperazone/sulbactam for the management of pulmonary infection, recombinant human brain natriuretic peptide to promote vasodilation and decrease cardiac workload, furosemide for diuresis, sacubitril/valsartan to reduce cardiac oxygen consumption, dapagliflozin to inhibit ventricular remodeling, spironolactone to prevent myocardial fibrosis and hypertrophy, and levosimendan to enhance cardiac contractility and facilitate vasodilation. Despite the absence of cardiovascular risk factors, the patient exhibited significantly elevated troponin levels and nonspecific ischemic changes on the electrocardiogram. To elucidate the etiology of the HF, coronary angiography was conducted once the patient condition stabilized to determine if coronary heart disease was present. The angiographic findings revealed dual vessel disease, characterized by 50% stenosis in both the right anterior descending and right distal coronary arteries; hence, no additional intervention was deemed necessary.

In view of the absence of known cardiovascular risk factors and an additional past medical history, but similar HF symptoms appeared repeatedly and not been effectively managed in the patient. In order to identify the etiology of HF, we once again inquiring about the detailed additional physical symptoms. Notably, the patient reported a 20-year history of dry eye syndrome, for which artificial tears were regularly employed, alongside symptoms of xerostomia. Consequently, we hypothesized the potential presence of SS. To substantiate this hypothesis, we firstly conducted an comprehensive analysis of the autoimmune antibody spectrum. The findings, and the results revealed the presence of ANA with a titer of 1:3200 (+++), HX >1:1000 (++), and positive results for both anti-SS-A and anti-SS-B antibodies (Table 1). Based on this finding, it was decided to proceed with a labial gland biopsy after taking the patient’ consent. Subsequent pathological findings of labial gland biopsy revealed small salivary gland tissues from the lower lip exhibiting interstitial infiltration by multifocal lymphocytes and plasma cells, with a lymphocyte density exceeding 50 per mm^2^ (Fig. 1D). According to the ACR/EULAR-approved diagnostic criteria of pSS,^[6]^ the patient meets the inclusion criteria and does not have any of the conditions listed as exclusion criteria, so she finally diagnosed with pSS (Table 2).

**Table 2:** The diagnosis of pSS of the patient according to ACR/EULAR-approved criteria.

Item	Weight/score	Patient score
Labial salivary gland with focal lymphocytic sialadenitis and focus score of $1 foci/4 mm^2^	3	3
Anti-SSA/Ro positive	3	**3**
Anti-SSA/Ro positive ocular staining score ≥ 5 (or van Bijsterveld score ≥4) in at least 1 eye	1	Not be determined
Schirmer test ≤5 mm/5 min in at least 1 eye	1	Not be determined
Unstimulated whole saliva flow rate ≤0.1 mL/min	1	Not be determined

ACR/EULAR = American College of Rheumatology and the European Alliance of Associations for Rheumatology, pSS = primary Sjögren’s syndrome, SSA = Sjögren's syndrome A.

Correspondingly, the treatment regimen was adjusted to include the immunosuppressant hydroxychloroquine and total glucosides of paeony capsules. Following discharge, the patient underwent ongoing treatment, which included hydroxychloroquine 20 mg once daily, Baishao capsules 20 mg once daily, furosemide 20 mg once daily, spironolactone 20 mg once daily, bisoprolol 5 mg once daily, valsartan 100 mg twice daily, dapagliflozin 10 mg once daily, and levosimendan 5 mg once daily. Throughout the one-year follow-up period, the symptoms of HF were effectively relieved, with no reported instances of readmission attributable to HF.

## 3. Discussion

SS is a systemic disease characterized by heterogeneous manifestations and the potential to cause multiorgan lesions, particularly affecting the salivary and lacrimal glands. Nearly all patients experience xerophthalmia and xerostomia, with data from a cohort of over 6000 patients indicating that 92% and 94% developed xerophthalmia and xerostomia, respectively.^[7]^ In addition to glandular symptoms, SS frequently presents with systemic manifestations. A multicenter study comprising 395 patients diagnosed with SS indicated that 30% of the cohort presented with systemic manifestations, including arthralgia or nonerosive arthritis (45%), pulmonary fibrosis (53%), interstitial lung disease (20%), peripheral nervous system symptoms (17%), cutaneous vasculitis (13%), and renal involvement such as tubulointerstitial nephritis and glomerulonephritis (10%).^[8–13]^ Consequently, the European Union of Rheumatology Associations (EULAR) formulated the EULAR SS Disease Activity Index to evaluate disease severity. This index encompasses 12 distinct domains: structural, lymphadenopathy, glandular, joint, skin, pulmonary, renal, muscular, peripheral nervous system, central nervous system, hematological, and biological.^[14]^ While the EULAR SS Disease Activity Index does not account for cardiovascular manifestations, a growing body of research suggests potential cardiac involvement in association with SS.^[15]^ Numerous studies have indicated that patients with SS exhibit elevated serum lipid levels, a higher incidence of atherosclerosis, and an increased risk of cardiovascular events compared to the general population.^[16]^ For example, a study by Zhang identified a higher incidence of pulmonary hypertension in patients with SS.^[17]^ Additionally, another study found that patients with SS are at an increased risk of subclinical atherosclerosis compared to healthy individuals.

To comprehensively assess the impact of SS on cardiovascular risk factors, various surrogate outcome indices have indicated that patients with SS exhibit increased carotid intima-media thickness,^[18]^ valve regurgitation and augmented left ventricular mass,^[19]^ pulmonary hypertension,^[20]^ as well as systolic dysfunction and reduced coronary reserve.^[21]^ These findings suggest that SS adversely affects cardiac structure and function. Although these manifestations are uncommon, failure to promptly identify them may result in missed or incorrect diagnoses within this patient population. The pathways through which SS contributes to HF encompass autoimmunity and persistent inflammatory processes. It is well-documented that immune complexes can cause damage to vascular endothelial cells,^[22]^ subsequently contributing to the development of atherosclerosis. Clinical investigations have demonstrated increased levels of serum Ro/SS-A antibodies and rheumatoid factors in individuals with SS.^[23]^ Furthermore, anti-Ro/SS-A and anti-La/SS-B antibodies have been linked to congenital heart block in neonates^[24]^; however, this correlation has not been substantiated in adult populations.

Several studies have demonstrated that adult patients with SS often exhibit prolonged P–R intervals, indicating a potential link to conduction system defects.^[25]^ Additionally, the chronic inflammatory state associated with SS is known to induce the production of various proinflammatory cytokines, such as interleukin (IL)-6, IL-8, and tumor necrosis factor alpha, which can result in myocardial damage and alterations in cardiac structure and wall thickness.^[26]^ Moreover, these proinflammatory cytokines may expedite the progression of atherosclerosis and contribute to the development of arrhythmias.

In this case study, a 70-year-old female patient, initially devoid of cardiovascular risk factors, presented with interstitial pneumonia and subsequently developed HF with preserved ejection fraction, leading to multiple hospital admissions over the past year. During the current hospitalization, she was additionally diagnosed with pulmonary hypertension and hypothyroidism. A comprehensive medical history indicated that the patient had experienced prolonged symptoms of xerostomia and xerophthalmia. Subsequent diagnostic evaluations, encompassing the Schirmer test, autoantibody profiling, and labial salivary gland biopsy, yielded positive findings consistent with the 2016 ACR/EULAR classification criteria for primary SS. Upon confirmation of the diagnosis, immunosuppressive therapy was initiated in conjunction with standard HF management, resulting in marked clinical improvements. A one-year follow-up revealed no additional hospitalizations due to HF. Consequently, we propose that the patient HF, interstitial pneumonia, pulmonary hypertension, and hypothyroidism may be associated with SS.

Traditionally, HF has been predominantly linked to ischemic cardiomyopathy, myocarditis, valvular heart disease, and hypertension, with comparatively limited attention given to autoimmune disorders, particularly SS. In this instance, prior therapeutic interventions concentrated exclusively on alleviating HF symptoms without addressing the underlying etiology, resulting in recurrent symptoms and misdiagnosis. This scenario often occurs due to the relatively nascent and underdeveloped status of rheumatology within the broader field of internal medicine, as numerous county-level and certain tertiary hospitals lack dedicated rheumatology departments or specialists. Furthermore, the insufficient understanding of SS among non-rheumatology specialists can result in misdiagnosis, particularly when the initial nonspecific symptoms are mistakenly attributed to menopause or diabetes-related conditions. Although cardiovascular manifestations of SS are infrequent, failure to detect them promptly may lead to missed or incorrect diagnoses within this patient population, potentially culminating in severe cardiovascular events.

This case underscores the critical importance of recognizing the systemic manifestations of SS and the necessity of conducting comprehensive patient histories and analyses in clinical practice. In instances of unexplained HF, it is imperative to adopt a proactive approach in identifying underlying etiologies to prevent reliance solely on symptomatic treatment. We propose the incorporation of autoantibody screening into the standard diagnostic protocol for unexplained HF to mitigate the risk of misdiagnosis.

## 4. Conclusion

In conclusion, this study suggests that the diagnosis and management of unexplained HF should adopt a holistic approach that transcends the isolated examination of individual factors. Considering that HF constitutes the terminal stage of various cardiac pathologies, its management should not be restricted to mere symptomatic relief. Instead, it is crucial to actively identify and address the underlying etiologies to achieve more effective therapeutic outcomes. In instances of unexplained and recurrent HF, there is a pronounced need to identify the underlying autoimmune diseases it’s sugge, such as pSS, as early as possible and highlights the efficacy of combining immunosuppressive therapy with conventional HF treatment as the optimal approach for managing such cases.

## Acknowledgments

We thank the patient for her collaboration.

## Author contributions

**Conceptualization:** Hongcai Zhang.

**Investigation:** Hongcai Zhang.

**Writing – original draft:** Jiao Wu, Wen Xie, Mingjun Han, Qian Nie.

**Writing – review & editing:** Hongcai Zhang, Wen Xie, Mingjun Han, Qian Nie.
